# COVID-19-Related Information Sources and the Relationship With Confidence in People Coping with COVID-19: Facebook Survey Study in Taiwan

**DOI:** 10.2196/20021

**Published:** 2020-06-05

**Authors:** Peng-Wei Wang, Wei-Hsin Lu, Nai-Ying Ko, Yi-Lung Chen, Dian-Jeng Li, Yu-Ping Chang, Cheng-Fang Yen

**Affiliations:** 1 Department of Psychiatry Kaohsiung Medical University Hospital Kaohsiung Medical University Kaohsiung Taiwan; 2 Department of Psychiatry School of Medicine College of Medicine, Kaohsiung Medical University Kaohsiung Taiwan; 3 Department of Psychiatry Ditmanson Medical Foundation Chia-Yi Christian Hospital Chia-Yi Taiwan; 4 Department of Senior Citizen Service Management Chia Nan University of Pharmacy and Science Tainan Taiwan; 5 Department of Nursing College of Medicine National Cheng Kung University Tainan Taiwan; 6 Department of Healthcare Administration Asia University Taichung Taiwan; 7 Department of Psychology Asia University Taichung Taiwan; 8 Graduate Institute of Medicine College of Medicine Kaohsiung Medical University Kaohsiung Taiwan; 9 Department of Addiction Science Kaohsiung Municipal Kai-Syuan Psychiatric Hospital Kaohsiung Taiwan; 10 School of Nursing The State University of New York University at Buffalo Buffalo, NY United States

**Keywords:** COVID-19, information, internet, coping, confidence, mental health, social media, Facebook, survey, online health information

## Abstract

**Background:**

People obtain information on the coronavirus disease (COVID-19) from the internet and other sources. Understanding the factors related to such information sources aids health professionals in educating individuals.

**Objective:**

This study used data collected from the online survey study on COVID-19 in Taiwan to examine what major COVID-19 information sources are available and which sources are significantly related to the self-confidence of people in coping with COVID-19 in Taiwan.

**Methods:**

A total of 1904 participants (1270 non–health-care workers and 634 health care workers) were recruited from the Facebook advertisement. Their major sources of information about COVID-19, the relationships between the sources and demographic factors, and the relationships between the sources and the self-confidence in coping with COVID-19 were surveyed.

**Results:**

Most Taiwanese people relied on the internet for COVID-19 information. Many respondents also used a variety of sources of information on COVID-19; such variety was associated with sex, age, and the level of worry toward COVID-19, as well as if one was a health care worker. For health care workers, the use of formal lessons as an information source was significantly associated with better self-confidence in coping with COVID-19. The significant association between receiving information from more sources and greater self-confidence was found only in health care workers but not in non–health-care workers.

**Conclusions:**

Medical professionals should consider subgroups of the population when establishing various means to deliver information on COVID-19.

## Introduction

The coronavirus disease (COVID-19) emerged in Wuhan, China at the end of 2019 and rapidly spread worldwide. The World Health Organization (WHO) declared the ongoing outbreak of COVID-19 as a public health emergency demanding global concern. Early in the outbreak, Taiwan was predicted to have the second highest number of COVID-19 cases due to the many people moving to and from Taiwan and China [1]. After all, Taiwan experienced the severe outbreak of the 2002-2003 severe acute respiratory syndrome (SARS), which originated from southern China; worldwide, Taiwan had the third highest number of SARS cases, after China and Hong Kong [2]. However, the experience with SARS made many Taiwanese people vigilant against COVID-19, which has aided in COVID-19 prevention.

For information on COVID-19, timeliness and accuracy—although difficult to attain and measure—are foundational to mitigating and curing the disease for both the public and the scientific community [3]. Among the public, the internet is the most popular source of information on the etiologies and intervention models of medical illnesses [4]. Information has been proliferating on traditional and social media since the COVID-19 outbreak [5]. A recent study of 21 countries found that the number of Google searches for “wash hands” increased with the lower speed of the COVID-19 spread [6]. Misinformation on COVID-19, however, has also been proliferating on the internet, especially on social media [7,8].

In addition to the internet, traditional media are also important sources of information during disease outbreaks. However, repeated media exposure to crisis-related information elevates anxiety and stress responses among people [9]. The public may also receive information on COVID-19 from medical staff and laypeople, such as their friends, family members, and coworkers. Because people obtain information on COVID-19 from various sources, understanding the factors related to such information sources aids health professionals in educating individuals in particular and the public at large. Thus, by developing information-delivery systems that are transparent and effective, self-confidence in coping with the pandemic can be improved in their audience.

The online survey study on COVID-19 in Taiwan (hereafter referred to as the Taiwan COVID-19 survey) was conducted to assess the life experiences of people in Taiwan during the COVID-19 outbreak. Generally, the online survey is a promising method for assessing how members of the public understand and perceive a fast-moving infectious disease outbreak [10]. This study used data collected from the Taiwan COVID-19 survey to examine the following two issues: (1) what major COVID-19 information sources are available as well as the relationships between these sources and demographic factors; and (2) which sources are significantly related to the self-confidence of people in coping with COVID-19? We were particularly interested in the information sources of traditional media and the internet with regard to advice on social distancing. Furthermore, because Taiwan’s Ministry of Health and Welfare recommends that health care workers should learn about COVID-19 through online or in-person lessons, we were also interested in whether the associations of information sources with people’s self-confidence differed between health care workers and non–health-care workers.

## Methods

### Participants

Participants were recruited through a Facebook advertisement from April 10 to April 20, 2020. Facebook users were eligible for this study if they were ≥20 years of age and living in Taiwan. The Facebook advertisement included a headline, main text, pop-up banner, and link to the research questionnaire website. We designed the advertisement to appear in the Facebook users’ “news feeds,” which is a continually updated list from advertisers and the user’s connections such as friends and the Facebook groups that they have joined. Our advertisement only targeted users’ news feeds as opposed to other Facebook advertising locations such as on the right column because news feed advertisements are most effective in recruiting research participants [11]. We targeted the advertisement to Facebook users by location (Taiwan) and language (Chinese), where Facebook’s advertising algorithm determined which users to show our advertisement to. To ensure that health care workers were recruited, we also posted the link of the Facebook advertisement to Line (a direct messaging app) and Facebook groups joined by health care workers.

This study was approved by the Institutional Review Board (IRB) of Kaohsiung Medical University Hospital (KMUHIRB-EXEMPT[I] 20200011). Because participation was voluntary and survey responses were anonymous, the IRB ruled that this study did not require informed consent. Our study participants were given no incentive for participation. We provided links to COVID-19 information from the Taiwan Centers for Disease Control, Kaohsiung Medical University Hospital, and Medical College of National Cheng Kung University for participants to learn more about COVID-19.

### Measures

In the survey, participants were asked about the following:

#### Sources of Information About COVID-19

We measured participants’ frequency of receiving COVID-19-related information from the following sources: the internet (including blogs, internet news, and social media, such as Facebook, Line, Twitter, and Plurk), friends, traditional media (including television, newspapers, and radio broadcasting), formal lessons on COVID-19 (whether online or in-person), medical staff in health care settings, coworkers, and family members. Participants rated their frequency for each source as never, sometimes, or frequently. Sources rated frequently were classified as major sources of information. The total numbers of sources of information about COVID-19 were summed.

#### Worry About COVID-19

We used the following question from the study of Liao et al [12] to assess how much respondents worried about COVID-19: “Please rate the level of your current worry toward COVID-19.” This question was rated from 1 (very mild) to 10 (very severe).

#### Self-Confidence in Coping With COVID-19

We used the following question adapted from the questionnaire on risk perception of an infectious disease outbreak [13] to assess how self-confident respondents were in coping with COVID-19: “How confident are you that you can cope well with COVID-19?” The question was rated from 1 (not confident at all) to 5 (very confident).

#### Demographic Variables

Data on respondents’ gender (female and male), age, and education level were also collected. Respondents who had high school qualifications or below and respondents who had university qualifications or above were classified into low and high education level groups, respectively. Respondents were also asked whether they were health care workers.

### Statistical Analysis

Data analysis was performed using SPSS 22.0 statistical software (IBM Corp). For each participant, the frequency of information-source use is expressed in terms of percentage. The associations of demographic characteristics and worry toward COVID-19 with COVID-19 information sources were examined using logistic regression analysis. The *P* value, odds ratio, and 95% CI were used to indicate significance. Because multiple comparisons were conducted, a *P* value of <.007 (.05/7) indicated significance. The factors related to the total number of major sources of information about COVID-19 were examined using multiple regression analysis. Moreover, the associations of each information source and total number of information sources with self-confidence were examined using multiple regression analyses as well as for the subgroups of health care workers and non–health-care workers. A two-tailed *P* value of <.05 indicated statistical significance.

## Results

In total, the data of 1904 respondents (1282 female and 622 male participants) were analyzed, with 70 of the original 1974 respondents excluded due to missing data. Table 1 presents the descriptive statistics for demographic characteristics, worry toward COVID-19, self-confidence in coping with COVID-19, and COVID-19 information sources. The mean age was 38.0 years (SD 10.8 years); most had a high education level; one-third were health care workers. The mean scores for worry and self-confidence were 6.1 (SD 2.2) and 3.1 (SD 0.8), respectively.

**Table 1:** Descriptive statistics for participants’ (N=1904) demographic characteristics, worry toward COVID-19, self-confidence in coping with COVID-19, and COVID-19 information sources.

Variables		Statistic	Range
**Gender, n (%)**			
	Female	1282 (67.3)	N/A^a^
	Male	622 (32.7)	N/A
Age (years), mean (SD)		38.2 (10.8)	20-74
**Education level, n (%)**			
	Low (high school or below)	212 (11.1)	N/A
	High (university or above)	1692 (88.9)	N/A
**Health care workers, n (%)**			
	No	1270 (66.7)	N/A
	Yes	634 (33.3)	N/A
Severity of worry toward COVID-19^b^, mean (SD)		6.1 (2.2)	1-10
Self-confidence in coping with COVID-19, mean (SD)		3.4 (0.8)	1-5
**Sources of information about COVID-19, n (%)**			
	Internet	1535 (80.6)	N/A
	Friends	403 (21.2)	N/A
	Traditional media	1018 (53.5)	N/A
	Academic lessons	391 (20.5)	N/A
	Medical staff	367 (19.3)	N/A
	Work colleagues	462 (24.3)	N/A
	Family members	475 (24.9)	N/A
**Number of information sources, n (%)**			
	0	200 (10.5)	N/A
	1	494 (25.9)	N/A
	2	453 (23.8)	N/A
	3	284 (14.9)	N/A
	4	196 (10.3)	N/A
	5	129 (6.8)	N/A
	6	66 (3.5)	N/A
	7	82 (4.3)	N/A
Total number of information sources, mean (SD)		2.4 (1.8)	0-7

^a^Not applicable.

^b^COVID-19: coronavirus disease.

The major source of information on COVID-19 in a high proportion of users was the internet, followed by traditional media, family members, coworkers, friends, formal lessons, and medical staff. Furthermore, more than one-quarter of participants received their COVID-19-related information from 1 source, followed by 2 sources, 3 sources, ≥5 sources, 0 sources, and 4 sources, with 2.4 sources as the mean (SD 1.8).

Table 2 presents the multiple regression results on the associations of demographic characteristics and worry toward COVID-19 with each information source. The results indicated that women were more likely to obtain information from traditional media and family members; older people were more likely to obtain information from traditional media and were less likely to obtain information from the internet and their work colleagues; and health care workers were more likely to obtain information from formal lessons, medical staff, and coworkers. For all information sources, except for formal lessons and medical staff, worry was significantly associated with information-source use.

**Table 2:** Factors related to COVID-19 information sources.

Variables	Internet		Friends		Traditional media		Academic lessons		Medical staff		Work colleagues		Family members	
	OR^a^ (95% CI)	*P* value	OR (95% CI)	*P* value	OR (95% CI)	*P* value	OR (95% CI)	*P* value	OR (95% CI)	*P* value	OR (95% CI)	*P* value	OR (95% CI)	*P* value
Males^b^	0.814 (0.638-1.040)	.10	1.008 (0.790-1.286)	.95	0.724 (0.594-0.883)	.001	0.939 (0.724-1.218)	.64	0.818 (0.618-1.084)	.16	0.741 (0.579-0.948)	.02	0.718 (0.566-0.911)	.006
Age	0.977 (0.967-0.988)	<.001	0.987 (0.976-0.998)	.02	1.016 (1.007-1.025)	<.001	1.000 (0.988-1.012)	>.99	0.991 (0.979-1.004)	.18	0.983 (0.972-0.994)	.002	0.997 (0.986-1.007)	.53
High educational level^c^	1.545 (1.097-2.176)	.01	1.016 (0.701-1.474)	.93	0.822 (0.608-1.111)	.20	0.995 (0.653-1.515)	.98	0.683 (0.441-1.058)	.09	0.706 (0.495-1.008)	.06	0.712 (0.515-0.983)	.04
Health care workers^d^	0.827 (0.647-1.057)	.13	1.309 (1.032-1.661)	.03	0.992 (0.814-1.210)	.94	5.891 (4.600-7.543)	<.001	10.645 (8.067-14.046)	<.001	3.500 (2.783-4.402)	<.001	0.827 (0.655-1.045)	.11
Worry toward COVID-19^e^	1.107 (1.052-1.165)	<.001	1.107 (1.051-1.165)	<.001	1.094 (1.049-1.140)	<.001	1.013 (0.959-1.069)	.64	1.065 (1.004-1.129)	.04	1.084 (1.031-1.141)	.002	1.102 (1.050-1.156)	<.001

^a^OR: odds ratio.

^b^Female as reference.

^c^High school or below as reference.

^d^Non–health-care worker as reference.

^e^COVID-19: coronavirus disease.

Table 3 presents the multiple regression results on the associations of demographic characteristics and worry toward COVID-19 with the number of information sources each respondent used. The results indicated that women and health care workers tended to use more information sources relative to their counterparts. Worry was also significantly associated with the number of information sources used.

**Table 3:** Factors related to number of COVID-19 information sources.

Variables	Beta	*t* test	*P* value
Males^a^	–.066	–2.909	.004
Age	–.037	–1.585	.11
High educational level^b^	–.024	–1.067	.29
Health care workers^c^	.117	5.218	<.001
Worry toward COVID-19^d^	.230	10.132	<.001

^a^Female as reference.

^b^High school or below as reference.

^c^Non–health-care worker as reference.

^d^COVID-19: coronavirus disease.

Table 4 presents the multiple regression results on the associations of each information source and the number of information sources used by a respondent with self-confidence for the health care worker and non–health-care worker subgroups. For health care workers, those who received information from formal lessons and from more sources had significantly greater self-confidence. For non–health-care workers, a nonsignificant association was noted between each information source and the total number of sources used by a participant with self-confidence.

**Table 4:** Associations of COVID-19 information sources with self-confidence in coping with COVID-19.

Variables	Non–health-care workers						Health care workers					
	Beta	*t* test	*P* value	Beta	*t* test	*P* value	Beta	*t* test	*P* value	Beta	*t* test	*P* value
Males^a^	.082	3.030	.002	.081	3.009	.003	.069	1.781	.08	.089	2.279	.02
Age	.002	0.088	.93	.002	0.054	.96	–.008	–0.205	.84	–.015	–0.401	.69
High educational level^b^	.052	1.902	.06	.050	1.854	.06	–.001	–0.039	.97	.014	0.361	.72
Worry toward COVID-19^c^	–.307	–11.258	<.001	–.309	–11.363	<.001	–.323	–8.525	<.001	–.333	–8.747	<.001
Information from the internet	.001	0.051	.96	N/A^d^	N/A	N/A	.064	1.552	.12	N/A	N/A	N/A
Information from friends	–.027	–0.906	.37	N/A	N/A	N/A	.019	0.435	.66	N/A	N/A	N/A
Information from traditional media	–.022	–0.767	.44	N/A	N/A	N/A	–.072	–1.628	.10	N/A	N/A	N/A
Information from academic lessons	.049	1.659	.097	N/A	N/A	N/A	.144	2.969	.003	N/A	N/A	N/A
Information from medical staff	.024	0.782	.44	N/A	N/A	N/A	.085	1.608	.11	N/A	N/A	N/A
Information from work colleagues	–.016	–0.492	.62	N/A	N/A	N/A	–.081	–1.644	.10	N/A	N/A	N/A
Information from family members	.052	1.703	.09	N/A	N/A	N/A	–.042	–0.986	.32	N/A	N/A	N/A
Total number of information sources	N/A	N/A	N/A	.035	1.296	.20	N/A	N/A	N/A	.078	2.041	.04

^a^Female as reference.

^b^High school or below as reference.

^c^COVID-19: coronavirus disease.

^d^Not applicable.

## Discussion

### Principal Findings

This online survey study found that most Taiwanese people relied on the internet for COVID-19 information, followed by traditional media. Many respondents also used a variety of sources of information on COVID-19; such variety was associated with sex, age, and the level of worry toward COVID-19, as well as if one was a health care worker. For health care workers, the use of formal lessons as an information source was significantly associated with better self-confidence in coping with COVID-19. The significant association between receiving information from more sources and greater self-confidence was found only in health care workers but not in non–health-care workers.

### The Internet and Traditional Media as COVID-19 Information Sources

Approximately 80% of participants received COVID-19 information online. The internet makes information on COVID-19 more accessible, especially for those staying indoors due to the pandemic, with the websites of official public health organizations being the highest-quality source of online information on COVID-19 and how to prevent it [14]. We found that age was positively and negatively related with receiving COVID-19 information from traditional media and from the internet, respectively. This result suggests a cohort effect on the information source related to major health issues. Because the internet is a popular and accessible information source, medical professionals should consider subgroups of the population, especially with respect to internet access, when delivering information online. Despite the popularity and accessibility of the internet, we did not find a significant association between use of the internet as an information source on COVID-19 and self-confidence in coping with COVID-19. Misinformation on COVID-19 is rife, especially on social media. The WHO and Taiwan’s Ministry of Health and Welfare made efforts to dispel such misinformation [7,8].

### Sources of Information About COVID-19 Among Health Care Workers

Because Taiwan’s Ministry of Health and Welfare recommended health care workers attend formal lessons (whether online or in-person) on COVID-19, they were more likely to receive COVID-19 information from formal lessons, which resulted in greater self-confidence. A study in China reported depression, anxiety, insomnia, and distress in a high proportion of health care workers who were never exposed to information on COVID-19 [15]. It was the uncertainty surrounding COVID-19, in addition to physical and psychological exhaustion, that resulted in such mental health problems [16]. Thus, our study and previous studies have demonstrated the necessity of providing timely and transparent formal lessons on COVID-19 for health care workers.

### Worry About COVID-19 and Sources of Information

We noted that more severe worry about COVID-19 was significantly associated with using more information sources. More sources of information about COVID-19 were also associated with higher self-confidence to cope with COVID-19 in health care workers. Overall, these results imply the benefits of using multiple information sources during the COVID-19 pandemic. However, the direction of the association is unclear: greater self-confidence can lead one to access information from multiple sources. Clarifying this direction of association warrants further study.

### Limitations

This study has some limitations. First, although recruiting participants through Facebook is a promising research method to target the public during fast-moving infectious disease outbreaks [10], Facebook users may not be representative of the population. A review of a study that recruited participants through Facebook reported a bias in favor of women, young adults, and people with higher education and incomes [17]. Second, the cross-sectional design of this study limited causal inference between sources of information, worry about COVID-19, and self-confidence. Third, this study was conducted during the period of COVID-19 mitigation but not during the period when COVID-19 first emerged in Taiwan.

### Conclusion

People may use a variety of sources to search for information on COVID-19, and various sources of information had various relationships with the confidence in coping with COVID-19. Medical professionals should consider subgroups of the population when establishing the various means to deliver information on COVID-19.

## Abbreviations

COVID-19: coronavirus disease
IRB: Institutional Review Board
SARS: severe acute respiratory syndrome
WHO: World Health Organization

## References

[1] (2020). Science and Engineering at Johns Hopkins.

[2] (2004). World Health Organization.

[3] Hua J, Shaw R (2020). Corona virus (COVID-19) "infodemic" and emerging issues through a data lens: the case of China. Int J Environ Res Public Health.

[4] National Research Council (2000). Networking Health: Prescriptions for the Internet.

[5] Kouzy R, Abi Jaoude J, Kraitem A, El Alam MB, Karam B, Adib E, Zarka J, Traboulsi C, Akl EW, Baddour K (2020). Coronavirus goes viral: quantifying the COVID-19 misinformation epidemic on Twitter. Cureus.

[6] Lin Y, Liu C, Chiu Y (2020). Google searches for the keywords of "wash hands" predict the speed of national spread of COVID-19 outbreak among 21 countries. Brain Behav Immun.

[7] Cuan-Baltazar J, Muñoz-Perez MJ, Robledo-Vega C, Pérez-Zepeda MF, Soto-Vega E (2020). Misinformation of COVID-19 on the internet: infodemiology study. JMIR Public Health Surveill.

[8] Bastani P, Bahrami M (2020). COVID-19 related misinformation on social media: a qualitative study from Iran. J Med Internet Res.

[9] Garfin D, Silver R, Holman E (2020). The novel coronavirus (COVID-2019) outbreak: amplification of public health consequences by media exposure. Health Psychol.

[10] Geldsetzer P (2020). Use of rapid online surveys to assess people's perceptions during infectious disease outbreaks: a cross-sectional survey on COVID-19. J Med Internet Res.

[11] Ramo DE, Rodriguez TMS, Chavez K, Sommer MJ, Prochaska JJ (2014). Facebook recruitment of young adult smokers for a cessation trial: methods, metrics, and lessons learned. Internet Interv.

[12] Liao Q, Cowling B, Lam W, Ng D, Fielding R (2014). Anxiety, worry and cognitive risk estimate in relation to protective behaviors during the 2009 influenza A/H1N1 pandemic in Hong Kong: ten cross-sectional surveys. BMC Infect Dis.

[13] (2015). Effective Communication in Outbreak Management for Europe.

[14] Hernández-García I, Giménez-Júlvez T (2020). Assessment of health information about COVID-19 prevention on the internet: infodemiological study. JMIR Public Health Surveill.

[15] Lai J, Ma S, Wang Y, Cai Z, Hu J, Wei N, Wu J, Du H, Chen T, Li R, Tan H, Kang L, Yao L, Huang M, Wang H, Wang G, Liu Z, Hu S (2020). Factors associated with mental health outcomes among health care workers exposed to coronavirus disease 2019. JAMA Netw Open.

[16] Neto MLR, Almeida HG, Esmeraldo JD, Nobre CB, Pinheiro WR, de Oliveira CRT, Sousa IDC, Lima OMML, Lima NNR, Moreira MM, Lima CKT, Júnior JG, da Silva CGL (2020). When health professionals look death in the eye: the mental health of professionals who deal daily with the 2019 coronavirus outbreak. Psychiatry Res.

[17] Whitaker C, Stevelink S, Fear N (2017). The use of Facebook in recruiting participants for health research purposes: a systematic review. J Med Internet Res.

